# Comorbidities and Complications in Parkinson’s Disease in Primary Care

**DOI:** 10.1111/ene.70697

**Published:** 2026-07-13

**Authors:** Olaitan Okunoye, Laura Horsfall, Louise Marston, Kate Walters, Anette Schrag

**Affiliations:** ^1^ Department of Clinical and Movement Neurosciences University College London London UK; ^2^ Institute of Health Informatics University College London London UK; ^3^ Department of Primary Care and Population Health University College London London UK

**Keywords:** comorbidity, complications, mortality, Parkinson's disease, primary care

## Abstract

**Background:**

Parkinson's disease (PD) is associated with an increased rate of mortality. It is unclear whether this is due to complications and comorbidities associated with the disease or the disease process of PD itself.

**Methods:**

We undertook a cohort study in patients with PD in an electronic primary care record database. Mortality rate ratios and risk differences per 1000 person‐years were calculated for each complication and comorbidity using multivariable Poisson regression analysis.

**Results:**

There were 10,104 patients with incident PD and 55,664 controls. The rate of falls, dementia, hallucinations, postural hypotension, stroke, depression, anxiety, sleep disorders, gastrointestinal and urinary disorders, headaches, epilepsy, and myocardial infarction was higher in individuals with PD compared to controls. Conversely, the rates of chronic obstructive pulmonary disease (COPD), congestive heart failure, and gout were lower. In the PD group, the comorbidities of dementia, falls, stroke, cancer, chronic heart failure, COPD, myocardial infarction, and gastrointestinal disorders were associated with higher mortality rates. However, for all comorbidities, the increase in mortality rate was similar to or lower in the PD group compared to people without PD, except for falls which tended to be associated with higher mortality in the PD group.

**Conclusion:**

This study provides data on the increased rate of mortality associated with different complications and comorbidities in PD. The mortality associated with these alone, however, does not explain the increased mortality in PD. This underscores the need for treatments to slow the underlying disease process.

## Graphical abstract

Many comorbidities are increased in PD. However, they are not associated with greater mortality than in controls, and are not sufficient to explain the increased mortality in PD.

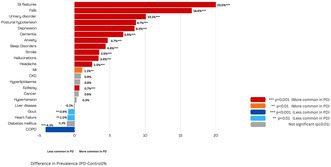

## 1 Introduction

Parkinson's disease (PD) is a chronic and slowly progressive disorder which leads to increasing disability over time, poor quality of life [1] and reduced life expectancy [2, 3]. A number of comorbidities and complications have been reported to be increased in people with PD compared to the general population including diabetes mellitus type II, epilepsy, dementia, depression, anxiety and hypertension which can be associated with worse outcomes [4, 5, 6, 7, 8]. It is not known whether the presence of any of these comorbidities is responsible for the increased mortality in PD. Data from previous studies regarding predictors of mortality are conflicting. Age and dementia are the most widely reported predictors of mortality in PD; however, there is considerable heterogeneity in methodology, selection of study population and results [2]. Many studies used hospital‐based data rather than population‐based data, which may not be representative of the general population and may be skewed towards more severe cases [9, 10, 11, 12, 13]. There are few population‐based studies which have examined multiple comorbidities and complications as predictors of mortality in people with PD.

In this study we therefore examined a range of comorbidities and complications to determine the effect of comorbidities in PD on mortality both in people with PD and in controls and examined whether they differentially affect mortality rates in patients with PD and controls.

## 2 Methods

### 2.1 Data Source

This was a cohort study of patients with PD in a primary care record, The Health Improvement Network (THIN), one of the largest databases containing anonymized electronic medicals records generated from more than 700 general practices and about 12 million patients' data from all over the UK [14] (3.7 million active patients), equivalent to 75.6 million patient years of data, covering 6.2% of UK population. All data are anonymised, processed and validated by CSD Medical Research UK [14]. THIN has data on patient demographics, details of visits to the general practitioners (GPs), disease diagnosis, symptoms, management, prescribed medications and death [15, 16]. Records of death and date of death have been reported to be reliable [17]. Townsend quintile, a measure of social deprivation based of patients' postal (zip) code [18], referrals to secondary care and free text information are all available in THIN. The Read classification system, a hierarchical coding system is used by General Practitioners to enter codes for symptoms and specific diagnoses [19, 20, 21]. It is estimated that about 98% of the population of the UK are registered with a General Practice (GP) [22] and most remain registered from birth until death. Additionally, more than 90% of NHS contacts are in general practice. The data quality has also been demonstrated to be high in independent validation studies [23, 24].

This study included person‐year data after the latest of the dates that the practices met the quality assurance standards in THIN: acceptable computer usage (ACU) and acceptable mortality reporting (AMR) [25, 26]. The ACU date is the date after which the practice is confirmed to have an average of at least one medical record, one additional health record and two prescriptions per patient per year [26]. The AMR date is the date after which the practice is confirmed to have a rate of mortality which is close to what is anticipated for a practice with its demographic features. This is based on data from the Office for National Statistics [25].

The NHS Health Research Authority has approved the use of IQVIA Medical Research Data (NHS Research Ethics Committee reference 23/EM/0151) for medical and public health research. This includes sharing data with external researchers for approved studies under Data Sharing Agreements. Patients can opt out of their data being used beyond their personal care. Those who opt out from THIN or the IQVIA Medical Research Extraction Scheme will be excluded from future data extracts. IQVIA Medical Research Data includes data from THIN, a Cegedim Ltd. database, licenced by IQVIA. This study was approved by IQVIA Medical Research Scientific Review Committee in June 2019 (SRC Reference Number: 19THIN034).

### 2.2 Participants

All individuals aged 50 years and over who were actively registered with a general practice and contributing data between 1st of January 2006 and 31st of December 2016 were included. We excluded all individuals with a diagnosis of PD before the start of our follow‐up period. This cohort of people with incident PD was compared with up to 6 people without Parkinson's disease, matched using random stratified sampling by age group, gender, calendar year and general practice. These controls joined the cohort if they met all inclusion criteria for individuals in the comparator group on the date of matching (index date). All were followed up until they died, left the general practice or stopped contributing data in THIN or end of study (follow‐up) period, whichever was earlier.

### 2.3 Parkinson's Disease Case Definition

Using the Read code classification, a case of Parkinson's disease in THIN was defined by a diagnostic Read code and at least two prescriptions of any of the five classes of antiparkinsonian medication including Levodopa‐containing medications, Dopamine‐receptor agonists, Amantadine, Monoamine‐oxidase‐B inhibitors–rasagiline, and selegiline, and Catechol‐O‐methyl transferase inhibitors (entacapone and tolcapone). Patients were identified through a computer search using a diagnostic Read code list for Parkinson's disease and drug code list for antiparkinsonian medications which were generated using developed methods [20]. The earliest electronic health record of Parkinson's disease diagnostic Read code or drug code for antiparkinsonian drug prescription was considered the index date.

This method of identification of people with Parkinson's disease has been shown to have a validity of 90% in the General Practice Research Database (GPRD, another primary health care database) [27, 28] and used in a previous study [29].

### 2.4 Assessment of Comorbidities and Complications

Data on comorbidities at any time during the observation period, social deprivation and smoking were extracted from the patients' electronic records. The following comorbidities and complications which are potentially associated with increased mortality or have been reported to be associated with Parkinson's disease were investigated: depression, anxiety, dementia, headache, epilepsy, hyperlipidaemia, hypertension, diabetes mellitus (type 1 and 2), myocardial infarction, stroke, congestive heart failure (CHF), chronic obstructive pulmonary disease (COPD), chronic kidney disease (CKD), liver disease, cancer, gout, gastrointestinal dysfunction, urinary and sleep disorders, postural hypotension and falls. Read code lists used for the computer search of comorbidities were developed for each comorbidity and complication using previously developed methods [20]. In order to identify those with hypertension, Read codes and blood pressure readings > 140/90 were used.

### 2.5 Confounders

The level of social deprivation was measured by the Townsend quintiles which scored from 1 (least deprived) to 5 (most deprived). Smoking status was classified as non‐smokers, ex‐smokers, and smokers.

### 2.6 Mortality Reporting

The quality of mortality reports in THIN is ensured by the AMR date which is the year from which each general practice is expected to have records of mortality which are equivalent to that from national statistics [30]. This is derived by reviewing reports of trends in death recording for individual practices (contributing data to THIN) against reports of predicted numbers of deaths generated from the Office for National Statistics (ONS), accounting for the demographics of the practice [25, 30]. We used all practices which are considered to be reporting all‐cause mortality comparable to these statistics. Records of death are assigned a death date which we used to identify individuals who died during the follow‐up.

### 2.7 Statistical Analysis

We calculated the number and percentage of patients with each comorbidity in the groups with and without Parkinson's disease. We examined the impact of each of these comorbidities and complications on mortality in people with and without PD by calculating mortality rate and mortality rate ratios associated with each comorbidity separately in the PD and non‐PD group. We ran separate analyses for each comorbidity and complication, adding multiplicative interaction terms with PD status (adjusting for age, gender, calendar year, social deprivation, and smoking). We then compared the adjusted mortality rate ratios in those with PD with and without comorbidities using multivariable Poisson regression models with robust standard errors and age as time scale, and a significance level of *p* < 0.01. The goodness of fit to the Poisson distribution was checked using the deviance statistics and by fitting negative binomial models and comparing outputs. The model assumptions were met as the count of events followed a Poisson distribution without overdispersion (mean and variance of outcome were similar). Outputs from the negative binomial models were similar.

We also estimated the difference in risk of death with a comorbidity among people with PD compared to controls by calculating estimates on the (relative) ratio scale as well as estimates on the (absolute) risk difference scale for interpretation. We calculated the margins of response as adjusted mortality rates for the different groups while holding all other variables at their observed values. The delta method was then used to determine standard errors for marginal effects.

All statistical analyses were conducted using Stata version 16MP (Stata Corporation, College Station, Texas).

## 3 Results

### 3.1 Baseline Features

**FIGURE 1:**
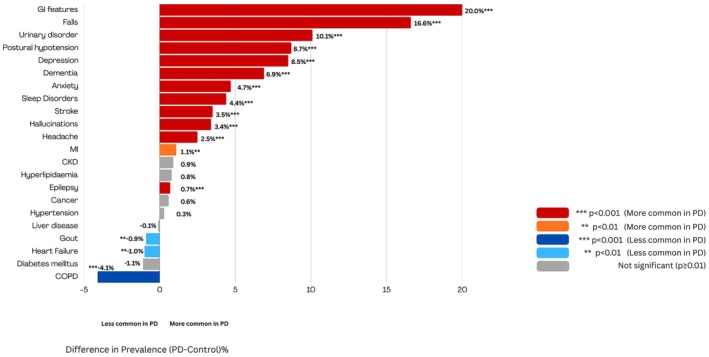
Differences in comorbidity prevalence between patients with Parkinson's disease and matched controls.

A total of 10,104 (incident cases) people had a new diagnosis of Parkinson's disease and were actively registered in THIN between the start of the study, 1st of January 2006 and end of study, 31st of December 2016. These were matched with 55,664 individuals who never had a record of Parkinson's disease diagnosis. There were 11,198 deaths in the cohort with 2031 in the Parkinson's disease group and 9167 deaths in the comparator group (Table 1). The number and percentage of patients with each comorbidity and complication are given in Figure 1. There was an increased rate of falls, dementia, hallucinations, postural hypotension, stroke, depression, anxiety, sleep, gastrointestinal and urinary disorders, headache, epilepsy and myocardial infarction in the PD group, and a decreased rate of chronic obstructive pulmonary disease (COPD), congestive heart failure and gout.

**TABLE 1:** Cohort characteristics.

	Parkinson's disease group *n* = 10,104	Non‐Parkinson's disease control group *n* = 55,664	*p* *
Age, years: median IQR	74.91 (62.26 to 87.56)	74.79 (61.9 to 87.68)	
Died, *n* (%)	2031 (20)	9167 (16)	< 0.001
Gender *n (%)*			
Men	6135 (60.72)	33,778 (60.68)	0.945
Women	3969 (39.28)	21,886 (39.32)	
Social deprivation, townsend quintiles *n* (%)			
1 (least deprived)	2811 (27.82)	13,746 (24.69)	< 0.001
2	2320 (22.96)	12,399 (22.27)	
3	1901 (18.81)	10,815 (19.43)	
4	1441 (14.26)	8728 (15.68)	
5 (most deprived)	895 (8.86)	5565 (10.0)	
Not recorded	736 (7.28)	4411 (7.92)	
Smoking			
Non‐smoker	5500 (54.43)	24,501 (44.02)	< 0.001
Ex‐smoker	3126 (30.94)	18,624 (33.08)	
Current smoker	690 (6.83)	7280 (13.08)	
Not recorded	788 (7.80)	5259 (9.45)	

* Chi‐squared test.

### 3.2 Mortality Associated With Comorbidities and Complications

As expected, the comorbidities of dementia, falls, stroke, cancer, chronic heart failure, COPD, myocardial infarction, and gastrointestinal disorders increased the adjusted mortality rate in people with PD.

However, none of the comorbidities increased mortality in patients with PD more than in the matched control group, except for falls (Tables 2, 3, 4, 5). On the contrary, the mortality in people with PD with some comorbidities was less increased than in people without PD, including dementia (Table 2), stroke (Table 3), cancer, chronic heart failure, COPD (Table 4). For example, COPD increased the adjusted mortality rate by 32.0 per 1000 person‐years (PYs) in people without PD and by 14.9 per 1000 PYs in people with PD (mortality rate difference 17.1 per 1000 PYs (95% CI 5.9 to 28.3); *p* = 0.003) (Table 4). Adjusting for smoking and social deprivation showed similar results.

**TABLE 2:** Mortality risk by PD status and comorbidity (neurological and psychiatric).

PD/Comorbidity Status	Unadjusted mortality rate (95% CI)	Adjusted mortality rate^a^ (95% CI)	Mortality rate ratio^b^ (95% CI; relative to controls without comorbidities)	Adjusted mortality rate difference (95% CI) between people with and without comorbidity	*p* for difference	Adjusted mortality rate difference (95% CI) between the PD and non‐PD groups due to comorbidity (Excess mortality in PD group)	*p* for difference
Anxiety							
PD^−^ Anxiety^−^	50.59 (49.52 to 51.69)	50.87 (49.08 to 52.66)	ref	−1.18 (−4.90 to 2.53)	0.53	3.53 (−4.84 to 11.91)	0.41
PD^−^ Anxiety^+^	44.93 (41.85 to 48.24)	49.69 (46.07 to 53.30)	0.96 (0.90 to 1.03)				
PD^+^ Anxiety^−^	56.56 (53.97 to 59.27)	56.28 (53.33 to 59.24)	ref	2.35 (−5.09 to 9.79)	0.54		
PD^+^ Anxiety^+^	53.10 (47.18 to 59.76)	58.63 (51.45 to 65.82)	1.05 (0.92 to 1.20)				
Depression							
PD^−^ Depression^−^	49.36 (48.24 to 50.51)	49.04 (47.28 to 50.80)	ref	9.6 6 (6.51 to 12.81)	< 0.001	−4.55 (−11.0 to 1.86)	0.16
PD^−^ Depression^+^	53.0 (50.66 to 55.43)	58.70 (55.48 to 61.93)	1.20 (1.14 to 1.27)				
PD^+^ Depression^−^	56.88 (54.03 to 59.88)	55.25 (52.17 to 58.32)	ref	5.12 (−0.59 to 10.83)	0.08		
PD^+^ Depression^+^	54.10 (49.85 to 58.70)	60.36 (55.06 to 65.66)	1.13 (1.04 to 1.24)				
Dementia							
PD^−^ Dementia^−^	43.48 (42.49 to 44.48)	46.59 (44.99 to 48.19)	ref	32.47 (27.55 to 37.40)	< 0.001	−16.88 (−24.43 to −9.33)	< 0.001
PD^−^ Dementia^+^	129.47 (123.47 to 135.36)	79.06 (73.98 to 84.14)	1.70 (1.59 to 1.81)				
PD^+^ Dementia^−^	50.12 (47.65 to 52.72)	53.26 (50.28 to 56.24)	ref	15.59 (9.72 to 21.47)	< 0.001		
PD^+^ Dementia^+^	84.63 (77.71 to 92.16)	68.86 (63.20 to 74.51)	1.32 (1.20 to 1.45)				
Headache							
PD^−^ Headache^−^	52.78 (51.65–53.94)	52.30 (50.46 to 54.13)	ref	−11.56 (−14.55 to −8.58)	< 0.001	9.16 (−1.65 to 16.67)	0.02
PD^−^ Headache^+^	34.95 (32.82 to 37.22)	40.73 (37.87 to 43.59)	0.77 (0.72 to 0.82)				
PD^+^ Headache^−^	57.58 (54.94 to 60.35)	56.94 (53.96 to 59.91)	ref	−2.40 (−9.38 to 4.57)	0.50		
PD^+^ Headache^+^	48.29 (43.01 to 54.22)	54.53 (47.83 to 61.24)	0.96 (0.84 to 1.09)				
Epilepsy							
PD^−^ Epilepsy^−^	49.34 (48.32 to 50.38)	50.08 (48.38 to 51.78)	ref	31.67 (21.84 to 41.50)	< 0.001	−13.53 (−31.60 to 4.55)	0.14
PD^−^Epilepsy^+^	84.71 (75.91 to 94.54)	81.76 (71.73 to 91.78)	1.60 (1.43 to 1.78)				
PD^+^Epilepsy^−^	55.49 (53.08 to 58.00)	56.06 (53.22 to 58.91)	Ref	18.14 (2.75 to 33.54)	0.02		
PD^+^Epilepsy^+^	75.75 (60.67 to 94.57)	74.21 (58.83 to 89.58)	1.35 (1.09 to 1.68)				
Hallucinations							
PD^−^ Hallucination^−^	49.53 (48.52 to 50.56)	49.99 (48.28 to 51.69)	Ref	24.7 (10.45 to 38.95)	0.0006	−20.97 (−38.35 to −3.60)	0.02
PD^−^ Hallucination^+^	124.77 (107.07 to 145.41)	74.69 (60.41 to 88.96)	1.49 (1.23 to 1.81)				
PD^+^ Hallucination^−^	55.57 (53.13 to 58.12)	56.78 (53.84 to 59.71)	Ref	3.73 (−6.0 to 13.45)	0.452		
PD^+^ Hallucination^+^	64.75 (54.41 to 77.05)	60.50 (50.89 to 70.11)	1.21 (1.03 to 1.42)				

Abbreviations: ^−^, absence of comorbidity; ^+^, presence of comorbidity; DM, Diabetes mellitus; HL, Hyperlipidaemia; HTN, Hypertension.

^a^ Adjusted for age, sex, calendar year and all other comorbidities.

^b^ Multivariable Poisson regression models.

**TABLE 3:** Mortality risk by PD status and comorbidity (vascular disease and risk factors).

PD/Comorbidity Status	Unadjusted mortality rate (95% CI)	Adjusted mortality rate^a^ (95% CI)	Mortality rate ratio^b^ (95% CI; relative to controls without comorbidities)	Adjusted mortality rate difference (95% CI) between people with and without comorbidity	*p* for difference	Adjusted mortality rate difference (95% CI) between the PD and non‐PD groups due to comorbidity (Excess mortality in PD group)	*p* for difference
Stroke							
PD^−^ Stroke^−^	44.06 (43.04 to 45.10)	47.31 (45.68 to 48.93)	Ref	20.02 (16.59 to 23.46)	< 0.001	−10.87 (−17.56 to −4.18)	0.001
PD^−^ Stroke^+^	93.15 (89.24 to 97.24)	67.33 (63.58 to 71.08)	1.44 (1.37 to 1.52)				
PD^+^ Stroke^−^	50.80 (48.33 to 53.40)	54.65 (51.64 to 57.67)	Ref	9.15 (3.30 to 15.0)	0.002		
PD^+^ Stroke^+^	83.81 (76.66 to 91.62)	63.81 (58.21 to 69.40)	1.16 (1.05 to 1.29)				
Myocardial infarction (MI)							
PD^−^ MI^−^	44.18 (43.13 to 45.26)	47.41 (45.68 to 49.15)	Ref	14.93 (12.14 to 17.73)	< 0.001	−7.79 (−14.07 to −1.51)	0.02
PD^−^ MI^+^	77.20 (74.25 to 80.28)	62.35 (59.41 to 65.28)	1.32 (1.26 to 1.38)				
PD^+^ MI^−^	51.25 (48.73 to 53.90)	54.92 (51.92 to 57.92)	Ref	7.14 (1.48 to 12.80)	0.01		
PD^+^ MI^+^	77.24 (70.87 to 84.19)	62.06 (56.61 to 67.51)	1.13 (1.02 to 1.24)				
Diabetes mellitus (DM)							
PD^−^ DM^−^	48.23 (47.11 to 49.38)	49.09 (47.26 to 50.91)	Ref	7.85 (5.07 to 10.63)	< 0.001	−1.69 (−8.64 to 5.27)	0.63
PD^−^ DM^+^	56.86 (54.54 to 59.28)	56.94 (54.17 to 59.70)	1.13 (1.08 to 1.19)				
PD^+^ DM^−^	54.94 (52.31 to 57.69)	55.38 (52.41 to 58.31)	Ref	6.16 (−0.08 to 12.40)	0.05		
PD^+^ DM^+^	60.69 (55.22 to 66.70)	61.55 (55.52 to 67.58)	1.08 (0.97 to 1.19)				
Hypertension (HTN)							
PD^−^ HTN^−^	49.02 (47.81 to 50.26)	53.80 (51.75 to 55.86)	1.0 (ref)	−8.26 (−10.59 to −5.93)	< 0.001	5.76 (0.04 to 11.48)	0.05
PD^−^ HTN^+^	52.36 (50.52 to 54.26)	45.54 (43.50 to 47.59)	0.85 (0.81 to 0.89)				
PD^+^ HTN^−^	52.15 (49.42 to 55.03)	57.48 (54.18 to 60.78)	1.0 (ref)	−2.50 (−7.44 to 2.44)	0.32		
PD^+^ HTN^+^	65.38 (60.72 to 70.41)	54.98 (50.66 to 59.31)	0.97 (0.88 to 1.05)				
Hyperlipidaemia (HL)							
PD^−^ HL^+^	52.23 (51.07 to 53.41)	52.09 (50.16 to 54.01)	1.0 (ref)	−7.24 (−9.96 to −4.53)	< 0.001	9.43 (−2.81 to 16.05)	0.01
PD^−^ HL^+^	41.22 (39.18 to 43.38)	44.84 (42.44 to 47.24)	0.84 (0.79 to 0.88)				
PD^+^ HL^−^	56.56 (53.91 to 59.34)	56.21 (53.31 to 59.11)	ref	2.18 (−3.99 to 8.36)	0.49		
PD^+^ HL^+^	53.92 (48.67 to 59.72)	58.39 (52.25 to 64.54)	1.00 (0.90 to 1.12)				

Abbreviations: ^−^, absence of comorbidity; ^+^, presence of comorbidity; DM, Diabetes mellitus; HL, Hyperlipidaemia; HTN, Hypertension.

^a^ Adjusted for age, sex, calendar year and all other comorbidities.

^b^ Multivariable Poisson regression models.

**TABLE 4:** Mortality risk by PD status and comorbidity (other).

PD/Comorbidity Status	Unadjusted mortality rate^a^ (95% CI)	Adjusted mortality rate (95% CI)	Mortality rate ratio^b^ (95% CI; relative to controls without comorbidities)	Adjusted mortality rate difference (95% CI) between people with and without comorbidity	*p* for difference	Adjusted mortality rate difference (95% CI) between the PD and non‐PD groups due to comorbidity (Excess mortality in PD group)	*p* for difference
Gout							
PD^−^ Gout^−^	49.42 (48.36 to 50.50)	50.90 (49.09 to 52.70)	ref	−1.15 (−4.55 to 2.26)	0.51	3.27 (−5.59 to 12.13)	0.47
PD^−^ Gout^+^	56.06 (52.70 to 59.64)	49.75 (46.44 to 53.06)	0.96 (0.91 to 1.02)				
PD^+^ Gout^−^	55.27 (52.80 to 57.85)	56.40 (53.52 to 59.28)	ref	2.12 (−6.04 to 10.29)	0.61		
PD^+^ Gout^+^	65.27 (56.58 to 75.30)	58.53 (50.38 to 66.67)	1.01 (0.86 to 1.16)				
Cancer							
PD^−^ Cancer^−^	39.52 (38.43 to 40.64)	43.63 (41.80 to 45.46)	ref	19.09 (16.69 to 21.49)	< 0.001	−11.44 (−17.17 to 5.71)	< 0.001
PD^−^ Cancer^+^	72.79 (70.63 to 75.02)	62.72 (60.30 to 65.15)	1.41 (1.35 to 1.47)				
PD^+^ Cancer^−^	49.27 (46.58 to 52.12)	53.75 (50.51 to 56.98)	ref	7.65 (2.53 to 12.76)	0.003		
PD^+^ Cancer^+^	70.62 (65.93 to 75.64)	61.39 (56.87 to 65.91)	1.13 (1.04 to 1.24)				
Chronic heart failure							
PD^−^ CHF^−^	44.75 (43.76 to 45.77)	47.16 (45.48 to 48.84)	ref	32.41 (27.79 to 37.03)	< 0.001	−12.76 (−22.88 to 2.65)	0.01
PD^−^ CHF^+^	116.02 (110.45 to 121.88)	79.57 (74.78 to 84.36)	1.71 (1.61 to 1.81)				
PD^+^ CHF^−^	52.31 (49.93 to 54.80)	54.68 (51.84 to 57.53)	1.0 (ref)	19.65 (10.14 to 29.15)	< 0.001		
PD^+^ CHF^+^	110.13 (97.50 to 124.40)	74.33 (64.89 to 83.77)	1.38 (1.20 to 1.58)				
Chronic obstructive pulmonary disease							
PD^−^ COPD^−^	45.86 (44.83 to 46.91)	47.02 (45.34 to 48.71)	ref	32.0 (27.69 to 36.29)	< 0.001	−17.13 (−28.33 to 5.94)	0.003
PD^−^ COPD^+^	85.18 (81.19 to 89.37)	79.01 (74.52 to 83.50)	1.69 (1.60 to1.78)				
PD^+^ COPD^−^	54.47 (52.04 to 57.01)	55.49 (52.64 to 58.34)	ref	14.86 (4.53 to 25.19)	0.005		
PD^+^ COPD^+^	78.90 (68.37 to 91.06)	70.34 (60.05 to 80.63)	1.25 (1.07 to 1.46)				
Chronic kidney disease (CKD)							
PD^−^ CKD^−^	43.02 (41.92 to 44.14)	50.0 (48.06 to 51.94)	ref	2.14 (−0.39 to 4.67)	0.1	2.32 (−3.03 to 7.67)	0.4
PD^−^ CKD^+^	69.76 (67.44 to 72.15)	52.14 (49.77 to 54.51)	1.02 (0.99 to 1.07)				
PD^+^ CKD^−^	48.67 (46.11 to 51.37)	55.10 (51.85 to 58.36)	1.0 (ref)	4.46 (−0.49 to 9.40)	0.08		
PD^+^ CKD^+^	77.89 (72.38 to 83.82)	59.56 (55.15 to 63.97)	1.10 (1.00 to 1.20)				
Liver disease (Liver ds)							
PD^−^ Liver ds^+^	49.75 (48.73 to 50.78)	50.38 (48.67 to 52.09)	ref	63.83 (40.49 to 87.18)	< 0.001	−26.03 (−74.17 to 22.10)	0.02
PD^−^ Liver ds^+^	94.13 (79.0 to 112.17)	114.22 (90.67 to 137.76)	2.34 (1.95 to 2.82)				
PD^+^ Liver ds^−^	55.85 (53.46 to 58.35)	56.36 (53.52 to 59.20)	ref	37.80 (−3.71 to 79.31)	0.07		
PD^+^ Liver ds^+^	88.41 (57.65 to 135.6)	94.16 (52.65 to 135.67)	1.64 (1.04 to 2.57)				

Abbreviations: ^−^, absence of comorbidity; ^+^, presence of comorbidity; DM, Diabetes mellitus; HL, Hyperlipidaemia; HTN, Hypertension.

^a^ Adjusted for age, sex, calendar year and all other comorbidities.

^b^ Multivariable Poisson regression models.

**TABLE 5:** Mortality risk by PD status and comorbidity (potential PD non‐motor manifestations and complications).

PD/Comorbidity Status	Unadjusted mortality rate (95% CI)	Adjusted mortality rate^a^ (95% CI)	Mortality rate ratio^b^ (95% CI; relative to controls without comorbidities)	Adjusted mortality rate difference (95% CI) between people with and without comorbidity	*p* for difference	Adjusted mortality rate difference (95% CI) between the PD and non‐PD groups due to comorbidity (Excess mortality in PD group)	*p* for difference
Gastrointestinal features (GIT)							
PD^−^ GIT^−^	41.29 (40.21 to 42.41)	45.77 (43.91 to 47.63)	ref	12.77 (10.03 to 15.51)	< 0.001	−6.03 (−11.19 to 0.86)	0.02
PD^−^ GIT^+^	71.93 (69.67 to 74.27)	58.55 (55.96 to 61.12)	1.28 (1.22 to 1.35)				
PD^+^ GIT^−^	47.41 (44.35 to 50.68)	53.33 (49.58 to 57.08)	ref	6.75 (2.04 to 11.45)	0.0049		
PD^+^ GIT^+^	64.85 (61.23 to 68.68)	60.08 (56.42 to 63.73)	1.11 (1.02 to 1.21)				
Urinary disorder (UD)							
PD^−^ UD^−^	48.23 (47.19 to 49.30)	49.54 (47.84 to 51.24)	ref	6.76 (2.07 to 11.44)	0.0047	−0.26 (−7.39 to 6.87)	0.94
PD^−^ UD^+^	68.71 (64.82 to 72.84)	56.30 (51.57 to 61.03)	1.14 (1.04 to 1.24)				
PD^+^ UD^−^	53.52 (50.90 to 56.26)	55.54 (52.41 to 58.67)	ref	6.50 (0.48 to 12.51)	0.034		
PD^+^ UD^+^	65.72 (60.19 to 71.76)	62.03 (56.47 to 67.60)	1.11 (1.00 to 1.22)				
Sleep disorder							
PD^−^ Sleep disorder^−^	49.55 (48.47 to 50.64)	50.0 (48.24 to 51.75)	ref	2.39 (−1.00 to 5.79)	0.17	1.60 (−6.0 to 9.20)	0.67
PD^−^ Sleep disorder^+^	54.04 (51.01 to 57.26)	52.39 (48.93 to 59.36)	1.05 (0.98 to 1.12)				
PD^+^ Sleep disorder^−^	55.98 (53.38 to 58.71)	56.37 (53.39 to 59.36)	ref	4.0 (−2.60 to 10.59)	0.24		
PD^+^ Sleep disorder^+^	56.50 (50.77 to 62.87)	60.37 (53.91 to 66.83)	1.07 (0.96 to 1.20)				
Postural hypotension (PH)							
PD^−^ PH^−^	48.63 (47.59 to 49.69)	50.09 (48.32 to 51.87)	ref	2.11 (−1.89 to 6.11)	0.302	−2.33 (−9.94 to 5.29)	0.55
PD^−^PH^+^	69.99 (65.48‐74.82)	52.20 (48.31 to 56.09)	1.04 (0.96 to 1.13)				
PD^+^ PH^−^	54.20 (51.65 to 56.89)	57.04 (53.95 to 60.13)	ref	−0.22 (−6.87 to 6.43)	0.95		
PD^+^ PH^+^	65.68 (59.44 to 72.57)	56.82 (50.59 to 63.05)	1.01 (0.90 to 1.14)				
Falls							
PD^−^ Falls^−^	43.74 (42.68 to 44.83)	49.01 (47.19 to 50.84)	ref	4.42 (1.60 to 7.24)	0.002	4.56 (−0.76 to 9.89)	0.01
PD^−^ Falls^+^	74.62 (71.90 to 77.43)	53.43 (50.70 to 56.17)	1.09 (1.03 to 1.15)				
PD^+^ Falls^−^	42.99 (40.38 to 45.77)	52.70 (49.13 to 56.27)	ref	8.99 (4.22 to 13.75)	0.0002		
PD^+^ Falls^+^	78.25 (73.66 to 83.13)	61.69 (57.81 to 65.57)	1.15 (1.05 to 1.26)				

Abbreviations: ^−^, absence of comorbidity; ^+^, presence of comorbidity; DM, Diabetes mellitus; HL, Hyperlipidaemia; HTN, Hypertension.

^a^ Adjusted for age, sex, calendar year and all other comorbidities.

^b^ Multivariable Poisson regression models.

## 4 Discussion

In this study, using a large general practice cohort, which is broadly representative of the general population in the UK, we found an increased rate of several comorbidities and complications in people with a diagnosis of PD, including falls, dementia, hallucinations, postural hypotension, stroke, depression, anxiety, sleep disorders, gastrointestinal and urinary disorders, headache, epilepsy, and myocardial infarction.

Several of the comorbidities increased the risk of death in patients with PD, in line with the known increase in mortality with these comorbidities in the general population, which was also seen in the control group. These included dementia, falls, stroke, cancer, chronic heart failure, COPD, myocardial infarction, and gastrointestinal disorders.

However, the mortality associated with these comorbidities did not increase the risk of death in people with PD over and above that in people without PD. On the contrary, the increase in mortality associated with some of these comorbidities was lower in patients with PD than in the non‐PD population.

There are no available comparable studies which have explored multiple comorbidities as predictors of mortality in PD. However, our results are contrary to most studies with varying study populations and designs which have focused on individual comorbidities as predictors of mortality in PD. For example, depression, dementia, and cardiovascular comorbidities have been reported to increase risk of death in PD [31, 32].

The increased mortality of these comorbidities was confirmed in this study, but it did not exceed that associated with these disorders in the non‐PD population. An important risk factor for these comorbidities is smoking status, and as patients with PD have reduced rates of smoking history, this may explain some of this difference. This may also apply to some other comorbidities such as cancer, stroke, and chronic heart failure.

There are two main findings from this study: (1) there is a mortality increase in patients with a number of comorbidities and complications in patients with PD compared to those without these comorbidities. These include dementia, falls, stroke, cancer, chronic heart failure, COPD, myocardial infarction and gastrointestinal disorders. Together with the increased prevalence of many of these comorbidities, the increased risk of death associated with these in PD and non‐PD populations emphasises the need to recognise, diagnose and treat the comorbidities in order to improve morbidity as well as mortality in PD; (2) The presence of comorbidities in patients with PD is not the key factor for increased mortality of PD, and it appears more likely that this is due to the disease process of PD itself as well as its complications leading to the increased mortality in PD, some of which are known to increase the risk of death [33, 34]. Disability in Parkinson's disease may also be significantly higher than that associated with comorbidities such as diabetes, heart disease or stroke in these patients, emphasising the huge burden [8] and potential mortality associated with PD itself. Despite treatment with antiparkinsonian medications, some symptoms remain difficult to treat [35]. or are not treatment‐responsive. However, it is also likely that the underlying disease process itself or its complications lead to the increased mortality of PD. In one study, the authors suggested that some features of PD such as hyposmia, freezing of gait and progression of the underlying pathology such as reduced dopamine transporter activity in the caudate could influence mortality in people with Parkinson's disease [36].

## 5 Strengths and Limitations

The main strength of this study is that this is a large population‐based sample in routine care, providing real‐life data on diagnoses of comorbidities and mortality. In addition, the large cohort of 10,104 people with Parkinson's disease provides sufficient power to detect comorbidities with small or large effects on mortality. We were also able to calculate multiple comorbidities and complications among people with Parkinson's disease and controls simultaneously in the same dataset. Whilst previous studies investigated small numbers of individual comorbidities or specifically investigated comorbidities and complications that are known to represent part of the spectrum of PD, we here investigated 21 common comorbidities, not all thought to be part of the PD presentation, but previously associated with increased mortality, and show their prevalence and potential prognostic value.

There are several limitations to this study. A potential bias in studies which use longitudinal data is differential misdiagnosis. It is possible that not all cases of PD received diagnostic confirmation from a specialist, and some might have had atypical parkinsonism which can become clear during later follow‐ups [37]. A further limitation to this study is that there are no measures of severity of Parkinson's disease in primary care. Since PD is a progressive neurodegenerative disease, a measure of disease severity or progression could be valuable to investigate the association of mortality with disease severity.

Furthermore, our cohort at entry may have been potentially influenced by varying healthcare seeking behaviours or health care access as those who have not come to medical attention may be missed. Population‐based studies where case identification relies only on medical records without population screening have been reported to result in some cases of PD being undiagnosed [38]. In spite of these limitations, THIN is reported to be representative of the UK population and has been validated and used in mortality/survival studies [39, 40]. Furthermore, this is not likely to have led to a differentially higher comorbidity rate in those with PD.

Future studies should be directed to investigating specific features of PD (with adequate follow‐up time) in addition to antiparkinsonian medications to examine their influence on mortality and how specialist care of these features can impact mortality.

## Author Contributions

**Olaitan Okunoye:** conceptualization, formal analysis, writing – original draft. **Kate Walters:** supervision, writing – review and editing. **Louise Marston:** formal analysis, writing – review and editing. **Laura Horsfall:** methodology, data curation, writing – review and editing. **Anette Schrag:** conceptualization, supervision, writing – review and editing.

## Conflicts of Interest

The authors declare no conflicts of interest.

## Data Availability

The data that support the findings of this study are available from The Health Improvement Network (THIN) database, licenced by IQVIA Medical Research Data. Restrictions apply to the availability of these data, which were used under licence for this study. Data are available from the author(s) with the permission of The data are available from the authors with permission of IQVIA Medical Research Data.
